# Determinants of research engagement in academic obstetrics and gynaecology

**DOI:** 10.1186/s12909-016-0640-2

**Published:** 2016-04-16

**Authors:** Ariadna Fernandez, Leslie Sadownik, Sarka Lisonkova, Geoffrey Cundiff, K. S. Joseph

**Affiliations:** Department of Obstetrics & Gynaecology, Faculty of Medicine, University of British Columbia, Room C427- 4500 Oak Street, Vancouver, BC V6H 3N1 Canada; Division of Gynaecologic Oncology, Department of Obstetrics & Gynaecology and Development & Educational Support, University of British Columbia, Vancouver, Canada; Division of Maternal Fetal Medicine, Department of Obstetrics & Gynaecology, University of British Columbia, Vancouver, Canada; Division of Gynaecologic Specialties, Department of Obstetrics & Gynaecology, University of British Columbia, Vancouver, Canada; School of Population and Public Health, University of British Columbia, Vancouver, Canada

**Keywords:** Obstetrics, Gynaecology, Attitude, Research involvement, Evidence-based practice

## Abstract

**Background:**

To identify the determinants of research engagement among faculty in an academic department of Obstetrics and Gynaecology.

**Methods:**

All members of the Department of Obstetrics and Gynaecology at the University of British Columbia were mailed an online version of the Edmonton Research Orientation Survey (EROS) in 2011 and in 2014. High scores on overall research engagement and on each of the 4 subscales, namely, value of research, value of innovation, research involvement and research utilization/evidence-based practice were quantified. Analyses were carried out on both surveys combined and on the 2014 survey separately. Logistic regression was used to identify determinants of high levels of research engagement.

**Results:**

The overall response rate was 37 % (130 responses; 54 respondents in 2011 and 76 respondents in 2014). The average EROS score was 140 (range 54 to 184) and 35 % of respondents had a score ≥150. Significant determinants of positive research engagement based on the overall EROS scale included being paid for research work (adjusted odds ratio [AOR] 22.1, 95 % confidence interval [CI] 2.47–197.7) and carrying out research during unpaid hours (AOR 6.41, 95 % CI 1.97–20.9). Age <50 years (AOR 11.0, 95 % CI 1.35–89.9) and clinical experience <20 years (AOR 19.7, 95 % CI 2.18–178.8) were positively associated, while journal reading during unpaid hours (AOR 0.21, 95 % CI 0.07–0.62) was negatively associated with specific EROS subscales.

**Conclusions:**

In a setting with a positive research orientation, research engagement among the faculty was associated with paid research time, research work and journal reading during unpaid hours and more recent entry into clinical practice.

## Background

Engagement of clinicians in research is essential for ensuring that relevant clinical questions are posed and addressed. Also, clinical experts sometimes have valuable insights that can lead to unexpected advances in medical science. The causal link between thalidomide and phocomelia was made simultaneously by an obstetrician, William Mcbride, and a pediatrician, Widukind Lenz, based on routine clinical observation [1–3]. Similarly, clinical insight obtained in routine practice led to the discovery that diethylstilbestrol use in pregnancy caused clear cell vaginal adenocarcinoma in daughters decades later [4, 5]. It is even claimed that obstetrician John Snow’s attribution of cholera epidemics to contaminated drinking water was based on a clinical conviction that was merely confirmed by subsequent epidemiologic research [6, 7].

Recognition of the clinician’s key role in advancing medical science notwithstanding, academic faculties and departments struggle with the challenge of effectively engaging clinical faculty in research [8–14]. There are many obstacles to clinician engagement and prominent among these challenges is the recruitment, training and retention of clinician researchers. Although a significant proportion of medical students express a strong interest in securing a full-time academic appointment with research involvement, resource issues hamper recruitment of clinician scientists. Specific issues include remuneration for research, anxiety regarding the competitive nature of research funding, inadequate research infrastructure and lack of protected time for research [8].

In 2010, the Department of Obstetrics and Gynaecology at the University of British Columbia created a Faculty Forum for Research dedicated to increasing clinician involvement in research. This effort is supported by a generous philanthropic donation, the Fred Bryans endowment. Given the goal of fostering an improved research culture within the department, we carried out a study exploring the determinants of positive research engagement among the faculty.

## Methods

In 2011 and 2014, the Edmonton Research Orientation Survey (EROS) was circulated to all faculty members within the Department of Obstetrics and Gynaecology at the University of British Columbia (UBC). The study received Research Ethics approval from the University of British Columbia/Children’s and Women’s Health Centre of British Columbia Research Ethics Board. The EROS is a validated tool developed to measure beliefs, attitudes and involvement in research [15–18]. The questionnaire consists of 38-items rated on a 5-point Likert scale from 1 (strongly disagree) to 5 (strongly agree). Four subscales measure respondents’ value of research, value of innovation, research involvement, and research utilization (i.e., evidence-based practice). EROS was developed to “assess the degree to which individual’s clinical practice is influenced by research findings” and the scale and its subscales are promising measures of research utilization and attitudes toward research [15, 16]. Each sub-scale is made-up of several items from the 38-point scale and is intended to measure various aspects of research orientation. Valuing Research pertains to a positive attitude towards research (e.g. “Even when funds are severely limited, it is important to support research activities.”); Research Involvement pertains to active participation in research (e.g. “I am actively involved in doing clinical research”; Being on the Leading Edge reflects value for innovation and change (e.g. “I am constantly looking for new information to help with my work.”; Evidence-Based Practice refers to whether or not respondents self-reported using research to guide their day to day practice (e.g.“ Reading the research literature has changed the way I practice.”) [16].

In 2011, we circulated the EROS survey via e-mail to all faculty members (*n* = 155), including two reminder e-mails. Address errors resulted in 10 emails being returned, and 61 completed responses were received (61/145; 42 %). In 2014, we recirculated the survey via e-mail to all faculty members (*n* = 208), followed by two reminder e-mails. No email address errors were encountered and 83 responses were received (83/208; 40 %). The responses from 2011 to 2014 were combined and analysed. Of the 144 survey questionnaires received, 14 were excluded from the analysis because the responses were substantially incomplete; thus a total of 130 responses were included in the study (overall response rate 130/353; 37 %).

We first analysed the 2011 and 2014 surveys separately in order to assess potential changes in research engagement over time. Since both surveys showed similar results, the responses from the 2 surveys were combined. However, the combined 2011 and 2014 survey analysis included the duplicated responses of some participants who would have responded to both surveys. The anonymized nature of the surveys prevented the identification of such respondents, and we attempted to assess potential distortion of our findings due to such duplication by comparing the results of the 2014 survey with the results of the combined 2011 and 2014 surveys. Sensitivity analyses present the results of the 2014 survey alone.

Characteristics of faculty members that were examined as potential determinants of research engagement included age, years of clinical experience, research degree, length of work week, journal reading during paid and unpaid hours and research work during paid and unpaid hours. For this analysis, we categorized all respondents into those who had high scores (i.e., scores of 4 or 5 on 30 or more of the 38 EROS items) or lower scores. Separate analyses identified determinants associated with high scores on each of the EROS subscales. Respondents who scored 4 or 5 on 6 or more of 8 items on the value of research subscale, 4 or more on the 6 items on the value of innovation subscale, 8 or more on the 10 items of the research involvement subscale and 5 or more on the 7 items of the research utilization/evidence-based practice subscale were considered to have a high score on that subscale. Logistic regression was used to quantify the independent association between each determinant and a high EROS score using adjusted odds ratios (AOR) and 95 % confidence intervals (CI). Analyses was first carried out for all items on the EROS combined and also separately for each of the EROS subscales. A 2 sided *P* value < 0.05 was considered statistically significant.

## Results

The overall response rate was 37 % (130 responses of 353; 54 respondents in 2011 and 76 respondents in 2014). The majority of respondents (54 %) were under 50 years of age and 58 % of faculty had less than 20 years of clinical experience (Table 1). A significant majority of respondents (72 %) had completed a course in statistics or research design; 52 % reported doing research work during unpaid hours; 21 % reported reading journals during unpaid hours; 35 % reported doing research during paid time and 34 % reported reading journals during paid time. Survey respondents were representative of all faculty members in the department in terms of type of position (clinical versus tenure track) and membership by division in General Obstetrics and Gynaecology, Gynaecologic Oncology, Gynaecologic Specialties, Maternal Fetal Medicine, and Reproductive Endocrinology and Infertility. The study population was also similar to the overall Departmental membership in terms of age (approximately 53 % of the Department members were <50 years of age). Details of other respondent characteristics are shown in Table 1.

**Table 1:** Characteristics of Edmonton Research Orientation Survey (EROS) respondents, Department of Obstetrics and Gynaecology, University of British Columbia

Characteristic	Number	Percent
	(*n* = 130)	
Age		
<50 years	70	53.8
Research degree		
MSc	21	16.2
PhD	12	9.2
No MSc or PhD	97	74.6
Research forum attendee		
Yes	57	44.5
Years of clinical experience		
<20	74	58.3
Attended research course		
Yes	93	72.1
Work hours per week		
<50	95	74.2
Journal reading during paid hours		
Yes	40	34.2
Journal reading during unpaid hours		
Yes	40	31.5
Research work during paid hours		
Yes	42	35.0
Research work during unpaid hours		
Yes	60	51.7
Specialty		
Clinician	30	23.1
Researcher	31	23.9
Administrator	7	5.4
Clinician specialist	46	35.4
Other	16	12.3
Do you have a current research project?		
Yes	76	58.5
Do you make conference presentations		
Yes	73	56.2
EROS score^a^		
<100	8	6.2
100–149	77	59.2
150–190	45	34.6

Subjects with missing responses not included (e.g., Journal reading during paid hours *n* = 117 and Research work during paid hours *n* = 120)

^a^ EROS score based on responses to 38 items each scored on a scale from 1 to 5

The average EROS score was 140 (standard deviation 24), with a range extending from 54 to 184 (maximum possible EROS score 190). Most respondents (59 %) has an EROS score between 100 and 149, 35 % had a score between 150 and 190 and a small fraction scored less than 100 (Table 1).

Table 2 shows the frequency of respondents with high EROS (scores of 4 or 5 on 30 or more of the 38 EROS items). High scores were more prevalent among those who had previously attended the research forum, did research work during paid hours or unpaid hours or were clinicians, administrators or others (i.e., relative to researchers). Work <50 h per week was associated with a higher scores, although this was of borderline significance. Having a Masters degree or a PhD degree was positively associated with a high score on the EROS, although neither association was statistically significant (Table 2).

**Table 2:** Proportion of survey respondents with high scores^a^ on the Edmonton Research Orientation Survey (EROS), Department of Obstetrics and Gynaecology, University of British Columbia

Characteristic	Total No.	Respondents with high scores^a^		Odds ratio	95 % CI	*P* value
		No.	%			
Age						
<50 years	70	24	34.3	1.13	0.51–2.51	0.75
>=50	60	19	31.7	1.00	(−)	-
Research degree						
MSc	21	8	38.1	1.27	0.68–2.38	0.46
PhD	12	6	50.0	1.67	0.88–3.18	0.19
No MSc or PhD	97	29	29.9	1.00	(−)	-
Research forum attendee						
Yes	57	26	45.6	2.66	1.77–6.08	0.01
No	71	17	23.9	1.00	(−)	-
Years of clinical experience						
<20	74	24	32.4	0.93	0.41–2.11	0.86
>=20	53	18	34.0	1.00	(−)	-
Attended research course						
Yes	93	35	37.6	2.11	0.80–5.69	0.10
No	36	8	22.2	1.00	(−)	-
Work hours per week						
<50	95	36	37.9	2.75	0.96–8.25	0.06
>=50	33	6	18.2	1.00	(−)	-
Journal reading during paid hours						
Yes	40	15	37.5	1.33	(0.55–3.18)	0.49
No	77	24	31.2	1.00	(−)	-
Journal reading during unpaid hours						
Yes	40	10	25.0	0.60	(0.24–1.50)	0.24
No	87	31	35.6	1.00	(−)	-
Research work during paid hours						
Yes	42	27	64.3	8.23	3.23–21.4	<0.0001
No	78	14	18.0	1.00	(−)	-
Research work during unpaid hours						
Yes	60	5	8.3	0.07	0.02–0.21	<0.0001
No	56	32	57.1	1.00	(−)	-
Specialty						
Clinician	30	16	53.3	5.94	1.58–23.7	0.002
Researcher	31	5	16.1	1.00	(−)	-
Administrator	7	4	57.1	6.93	1.05–60.7	0.04
Clinician specialist	46	9	19.6	1.26	0.33–4.97	0.70
Other	16	9	56.3	6.69	1.41–34.1	0.007
Do you have a current research project?						
Yes	76	28	36.8	1.52	0.67–3.47	0.28
No	54	15	27.8	1.00	(−)	-
Do you make conference presentations						
Yes	73	27	37.0	1.50	0.67–3.40	0.29
No	57	16	28.1	1.00	(−)	-

Subjects with missing responses not included (e.g., 13 subjects with missing values not included in Journal reading during paid hours and 10 subjects not included in Research work during paid hours, etc.)

^a^ Respondents with high scores were those scoring 4 or 5 on 30 or more of the 38 items of the EROS

Analyses restricting the study to the 2014 survey (76 responses) showed that high scores on the EROS were significantly associated with research work during paid hours (OR 20.0, 95 % CI 1.83–219.1, *P* value 0.01) and research work during unpaid hours (OR 8.77, 95 % 2.25–34.2, *P* value 0.002, Table 3 upper panel). These findings were similar to analyses involving all respondents and therefore subsequent analyses presented include respondents from both the 2011 and 2014 surveys (Table 3, lower panel). Research work during paid hours (AOR 22.1, 95 % CI 2.47–197.7, *P* value 0.006) and research work during unpaid hours (AOR 6.41, 95 % CI 1.97–20.9, *P* value 0.002) were significantly associated with high scores on the EROS in these analyses combining the 2011 and 2014 surveys. Supplementary analyses carried out after removing the 4 journal reading and research work variables from the model did not substantially alter the associations between the other determinants and high scores on the EROS, with the exception of work hours per week <50 (AOR 2.77, 95 % CI 0.99–7.75, *P* value 0.05).

**Table 3:** Logistic regression analysis showing respondent characteristics associated with high scores on the Edmonton Research Orientation Survey (all subscales), Department of Obstetrics and Gynaecology, University of British Columbia.

Characteristic	Adjusted odds ratio	95 % CI	*P* value
Respondents to 2014 survey only (*n* = 76)			
Age <50 years	6.11	0.49–75.5	0.16
Research degree: Yes	1.08	0.23–5.06	0.92
Years of clinical experience <20	10.6	0.78–144.9	0.08
Attended courses in research design: Yes	3.88	0.62–24.3	0.15
Work hours per week: <50	1.09	0.23–5.29	0.91
Journal reading during paid hours: Yes	0.12	0.01–1.34	0.08
Journal reading during unpaid hours: Yes	0.86	0.21–3.58	0.84
**Research work during paid hours: Yes**	**20.0**	**1.83–219.1**	**0.01**
**Research work during unpaid hours: Yes**	**8.77**	**2.25–34.2**	**0.002**
Respondents to 2011 and 2014 surveys (*n* = 111^a^)			
Age <50 years	1.74	0.40–7.63	0.47
Research degree: Yes	1.22	0.35–4.24	0.75
Years of clinical experience <20	4.15	0.81–21.4	0.09
Attended courses in research design: Yes	2.40	0.59–9.75	0.22
Work hours per week: <50	2.21	0.60–8.20	0.24
Journal reading during paid hours: Yes	0.15	0.02–1.34	0.09
Journal reading during unpaid hours: Yes	0.86	0.28–2.63	0.79
**Research work during paid hours: Yes**	**22.1**	**2.47–197.7**	**0.006**
**Research work during unpaid hours: Yes**	**6.41**	**1.97–20.9**	**0.002**

The upper panel shows results of the 2014 survey, while the lower panel shows the results of the surveys in 2011 and 2014^a^19 subjects had missing values in the 2011 survey for Journal reading during paid hours, Research work during paid hours, etc. and were not included in the regression model)

Statistically significant associations appear in bold text

Table 4 shows the factors associated with high scores on value of research and value of innovation subscale of EROS. Characteristics positively associated with a high score on the value of research subscale were research work during paid hours (AOR 5.76, 95 % CI 1.28–25.8, *P* value 0.02) and research work during unpaid hours (AOR 4.19, 95 % CI 1.37–12.8 *P* value 0.01). Journal reading during unpaid hours was negatively associated with high scores on the value of research subscale (AOR 0.21, 95 % CI 0.07–0.62, *P* value 0.005). None of the characteristics examined were significantly associated with high scores on the value of innovation subscale.

**Table 4:** Logistic regression analysis showing respondent characteristics associated with high scores on the ‘Value of Research’ subscale and on the ‘Value for Innovation’ subscale of the Edmonton Research Orientation Survey 2011 and 2014 (*n* = 111), Department of Obstetrics and Gynaecology, University of British Columbia

Characteristic	Adjusted odds ratio	95 % CI	*P* value
Value of Research			
Age <50 years	0.34	0.08–1.49	0.15
Research degree: Yes	0.34	0.10–1.14	0.08
Years of clinical experience <20	0.58	0.14–2.50	0.47
Attended courses in research design: Yes	1.58	0.54–4.67	0.40
Work hours per week: <50	0.55	0.18–1.69	0.30
Journal reading (during paid hours): Yes	1.71	0.47–6.28	0.41
**Journal reading (during unpaid hours): Yes**	**0.21**	**0.07–0.62**	**0.005**
**Research work (during paid hours): Yes**	**5.76**	**1.28–25.8**	**0.02**
**Research work (during unpaid hours): Yes**	**4.19**	**1.37–12.8**	**0.01**
Value for innovation			
Age <50 years	2.68	0.41–17.3	0.30
Research degree: Yes	0.51	0.10–2.58	0.42
Years of clinical experience <20	2.11	0.36–12.5	0.41
Attended courses in research design: Yes	2.35	0.60–9.18	0.22
Work hours per week: <50	2.23	0.58–8.60	0.25
Journal reading (during paid hours): Yes	3.97	0.48–33.2	0.20
Journal reading (during unpaid hours): Yes	0.43	0.12–1.55	0.20
Research work (during paid hours): Yes	0.85	0.07–9.64	0.89
Research work (during unpaid hours): Yes	3.88	0.52–28.8	0.19

Statistically significant associations appear in bold text

Table 5 shows associations between respondent characteristics and high scores on the research involvement and research utilization/evidence-based practice subscales of EROS. High scores on the research involvement subscale were positively associated with conducting research during paid hours (AOR 14.6, 95 % CI 2.90–92.9, *P* value 0.001) and conducting research during unpaid hours (AOR 6.48, 95 % CI 1.86–22.6, *P* value 0.003). The association between attending a course in research design or statistics and high scores on the research involvement subscale was borderline significant (AOR 4.91, 95 % CI 0.94–25.6, *P* value 0.06). The characteristics associated with a high score on the research utilization/evidence-based practice sub-scale were age <50 years, clinical experience of <20 years and conducting research during unpaid hours. Respondents <50 years of age were more likely to have a high score on this subscale (AOR 11.0, 95 % CI 1.35–89.8, *P* value 0.03), as were those with <20 years of clinical experience (AOR 19.7, 95 % CI 2.18–178.8, *P* value 0.008) and those doing research work during unpaid hours (AOR 8.15, 95 % CI 1.60–41.5, *P* value 0.01).

**Table 5:** Logistic regression analyses showing respondent characteristics associated with high scores on the ‘Research Involvement’ subscale and the ‘Research Utilization/Evidence-Based Practice’ subscale of the Edmonton Research Orientation Survey 2011 and 2014 (*n* = 111), Department of Obstetrics and Gynaecology, University of British Columbia

Characteristic	Odds ratio	95 % CI	*P* value
Research Involvement			
Age <50 years	1.42	0.26–7.86	0.69
Research degree: Yes	0.70	0.18–2.77	0.61
Years of clinical experience <20	0.94	0.16–5.47	0.94
Attended courses in research design: Yes	4.91	0.94–25.6	0.06
Work hours per week: <50	0.75	0.18–3.12	0.69
Journal reading (during paid hours): Yes	0.79	0.16–3.99	0.77
Journal reading (during unpaid hours): Yes	0.72	0.20–2.53	0.60
**Research work (during paid hours): Yes**	**14.6**	**2.90–72.9**	**0.001**
**Research work (during unpaid hours): Yes**	**6.48**	**1.86–22.6**	**0.003**
Research Utilization/Evidence-Based Practice			
**Age <50 years**	**11.0**	**1.35–89.8**	**0.03**
Research degree: Yes	0.33	0.07–1.50	0.15
**Years of clinical experience <20**	**19.7**	**2.18–178.8**	**0.008**
Attended courses in research design: Yes	3.18	0.79–12.7	0.10
Work hours per week: <50	2.12	0.48–9.36	0.32
Journal reading (during paid hours): Yes	1.40	0.18–10.7	0.75
Journal reading (during unpaid hours): Yes	0.76	0.23–2.53	0.65
Research work (during paid hours): Yes	1.21	0.15–10.2	0.86
**Research work (during unpaid hours): Yes**	**8.15**	**1.60–41.5**	**0.01**

Statistically significant associations appear in bold text

## Discussion

Our study showed a high level of positive research engagement among faculty, particularly among clinicians. In this setting, research work during paid and unpaid hours was associated with a high level of research engagement. Analyses focusing on the subscales of research engagement showed that value of research was positively associated with paid and unpaid research work and negatively associated with journal reading during unpaid hours. No significant associations were observed with value of innovation, while research involvement was associated with paid and unpaid research work, and research utilization/evidence-based practice was associated with age <50 years, years of clinical experience <20 and research during unpaid hours. Having attended a course in statistics or research design was positively associated with research involvement, although this association was of borderline significance.

Several of the findings of our study confirm that remuneration issues strongly influence research engagement among academic faculty. Although this issue has been previously identified, adequate research funding for clinical faculty remains an obstacle. On a positive note, doing research during unpaid time was associated with research engagement, suggesting that inadequate remuneration negatively impacts buts does not eliminate research engagement. Moreover, the higher level of research utilization/evidence-based practice among the younger respondents (as evidenced by the association with age <50 years and clinical experience <20 years) is a testament to the contemporary academic culture that has placed a premium on such issues from medical school onwards. The high level of research engagement among clinicians is also heartening, although this may potentially highlight our failure to adequately harness and translate this enthusiasm for research into research activity.

The negative, albeit non-significant, association between having a research degree and value of research was unexpected. One can speculate that this finding reflects frustration among clinicians with research degrees who feel unfulfilled because of time and other professional pressures. On the other hand, our study also showed a strong positive association (of borderline significance) between attending a course in statistics or research design and a high score on the research involvement subscale. The contrast between these findings is noteworthy and future studies should seek to clarify why these 2 markers of research commitment show diametrically opposite associations with subdomains of research engagement.

The strengths of our study included the use of a validated tool that is demonstrated to have high reliability and internal consistency [15, 16]. Our 37 % response rate was lower than ideal but higher than rates of 30–34 % typically reported for surveys among Canadian physicians (even though such rates vary widely) [19–21]. Nevertheless, the self-selected nature of survey respondents likely implies a possible overestimation in the relatively high rate of research engagement noted in our study. However, this self-selection bias is likely to have a more limited impact on the associations noted in our study. Another weakness of the study was the relatively small study size, which affected the precision of the estimates in logistic regression analyses.

## Conclusion

Our study provides some evidence to suggest that research engagement in an academic department is highly linked to remuneration issues. Other factors that also affect specific subdomains of research engagement include age <50 years, clinical experience <20 years and having attended a statistics or research design course. These factors highlight some of the complexities involved in fostering research in academic clinical departments. Further studies and innovative proposals are required to foster a research culture that can increase recruitment, training and retention of clinicians in research endeavours.

### Ethics approval and consent to participate

Research Ethics approval for this study was granted by The University of British Columbia/Children’s and Women’s Health Centre of British Columbia Research Ethics Board (UBC C&W REB), a UBC-affiliated Research Ethics Board (REB) for the Oak Street campus (http://www.cfri.ca/research-support/reb); reference number H15-00767.

### Consent for publication

Not applicable.

### Availability of data and materials

The data from this research was collected under quality assurance program of the departmental research program, and consent of participants for data sharing was not obtained. As per Research Ethics Board rules, the untabulated data cannot be publicly shared as consent for this was not obtained.

## Abbreviations

AOR: Adjusted Odds Ratios
CI: Confidence Intervals
EROS: Edmonton Research Orientation Survey
UBC: University of British Columbia
